# Undifferentiated pleomorphic sarcoma of the spleen: a case report and literature review

**DOI:** 10.1186/s40792-023-01734-4

**Published:** 2023-09-20

**Authors:** Raphael Gatt, Luca Casingena, David Pisani, Rachel Agius, Noel Cassar

**Affiliations:** Mater Dei Hospital, Msida, Malta

**Keywords:** Undifferentiated pleomorphic sarcoma, Malignant fibrous histiocytoma, Spleen, Splenectomy, Liver metastasectomy

## Abstract

**Background:**

Undifferentiated pleomorphic sarcoma is an uncommon sarcoma and its presence in the spleen is even rarer, with only a handful of cases reported in English literature. It is typically only diagnosed following histological analysis. Its rarity also means that there is little consensus over ideal management.

**Case presentation:**

This report presents a case of a 40-year-old Caucasian male who was found to have a splenic mass after presenting with non-specific abdominal pain and generalized malaise. Numerous imaging modalities were used which demonstrated a large partially solid and partially cystic lesion in spleen with no evidence of metastasis. As core biopsies were undiagnostic, he was planned for a diagnostic and therapeutic splenectomy. However, despite magnetic resonance imaging 11 days prior to his operation showed no evidence of liver metastasis, a massive splenic tumour with hepatic metastases was identified intraoperatively. An open splenectomy, distal pancreatectomy and liver metastasectomy was hence carried out. Histological analysis confirmed liver metastasis secondary to a splenic undifferentiated pleomorphic sarcoma. The patient recovered well and was discharged home. He presented again three weeks after his operation with lower back pain, abdominal pain and fever. Computed tomography demonstrated extensive recurrent disease burden in the peritoneum and liver. The patient passed away a month after surgery.

**Conclusion:**

Splenic undifferentiated pleomorphic sarcoma is a rare tumour which may pose a significant diagnostic challenge on both clinical and histopathological grounds. Following diagnosis and treatment, its aggressive nature often results in a poor prognosis. Current literature fails to delineate any superior management strategy to increase survival.

## Background

Soft tissue sarcomas (STS) are rare malignant tumours arising from mesenchymal tissues which make up around 2% of all adult malignancies [1]. Undifferentiated pleomorphic sarcoma (UPS), formerly known as malignant fibrous histiocytoma (MFH) or high-grade spindle cell sarcoma, is an aggressive form of soft tissue sarcoma which makes up 10–20% of STS [2]. It is commoner in males and frequently presents as a soft tissue tumour in the thigh and wall of the trunk. Whilst the occurrence of this tumour beyond these sites is extremely unusual, it has been documented in the head and neck, retroperitoneum and other internal organs including the spleen [3]. It is also one of the commonest radiation-induced sarcomas together with spindle cell sarcomas, osteosarcomas and fibrosarcomas [1, 4]. 

Given the disease’s prevalence of 0.08 to 1 per 100,000 people, little is known regarding the ideal management of such cases, having no established staging criteria or oncological treatments. The generally accepted treatment involves radical surgical resection followed by chemotherapy or radiotherapy, depending on the tumour size or the presence of metastatic disease. Despite such aggressive therapy, recurrence rates range after 5 years range between 10 and 42%, whilst 31 to 40% develop distant metastasis [5].

It remains a diagnostic challenge, being characterized by the absence of genetic characteristics and specific lines of differentiation on histological examination [6]. Pathological subtyping of these tumours into pleomorphic, myxoid, giant cell, inflammatory and angiomatoid variants is of no prognostic impact [7–9]. In addition, given the rarity of the disease in the spleen, it is frequently misdiagnosed on clinical and radiological grounds as lymphoma or metastatic malignancy. This case report and literature review is aimed at highlighting the clinical presentations, management, histological features and survival rates in order to aid further understanding of the clinical course and pathology of this rare entity.

## Case presentation

This report presents the case of a previously healthy 40-year-old Caucasian male with no history of malignancies or significant radiation exposure. He presented to the Emergency Department with an 8-day history of central epigastric pain which radiated to the back and was associated with generalized malaise and low-grade fever. Clinical examination demonstrated palpable splenomegaly of around 4 fingerbreadths below the left costal margin with an otherwise soft abdomen. He had a raised white cell count (WCC) of 24.3 × 10^9^/L, a haemoglobin level of 12.1 g/dL, a platelet count of 201 × 10^9^/L and a C-reactive protein (CRP) of 84.2 mg/L.

**Fig. 1:**
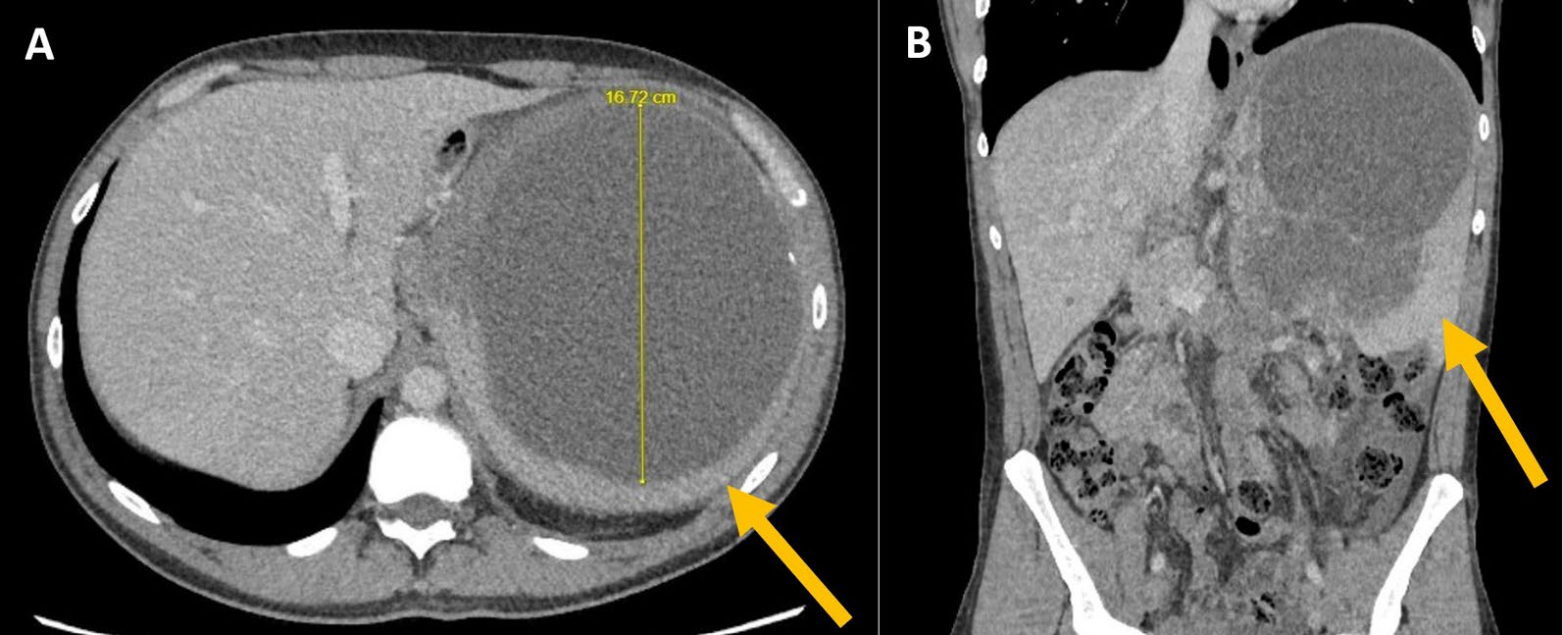
CT axial (**A**) and coronal view (**B**) demonstrating splenic size

Ultrasonographic (US) studies showed massive splenomegaly of 20.1 cm in diameter with a heterogeneous mass in splenic hilum measuring 12 cm by 9 cm. A homogeneous fluid collection was also noted adjacent to cranial aspect of spleen measuring 16.7 cm by 15 cm. The liver was normal on ultrasound studies. Computed tomographic (CT) imaging demonstrated a 16 cm × 14 cm × 21 cm partially solid partially cystic lesion in spleen with mass effect on surrounding structures, as seen in Fig. 1, with no evidence of distant metastases. Magnetic resonance (MR) imaging was also performed to further characterize the mass. Findings included a solid component towards the hilum and lower splenic pole, as well as an upper central cystic component containing hyperintense fluid (likely representing blood or mucin content) on T1 weighed images. Given that the hilar component was abutting the pancreatic tail, the possibility of the splenic mass representing direct infiltration by a distal pancreatic tumour (particularly an acinar cell carcinoma) was raised. The liver showed a few simple cysts, with no evidence of metastatic deposits on this scan.

An broad infectious screen, including human immuno-deficiency virus, hepatitis A, B and C, toxoplasma, rubella, cytomegalovirus, herpes simplex, Epstein–Barr virus, rickettsia, leishmaniosis, leptospirosis, mycoplasma and parvovirus, was negative.

Core needle biopsies of the spleen were attempted twice, both of which comprised entirely necrotic material with no viable tumour tissue present. Immunohistochemistry only demonstrated non-specific expression of CD10, along with a high Ki67 index. Whilst the possibility of a necrotic lymphoma was entertained, the biopsies were deemed non-diagnostic.

**Fig. 2:**
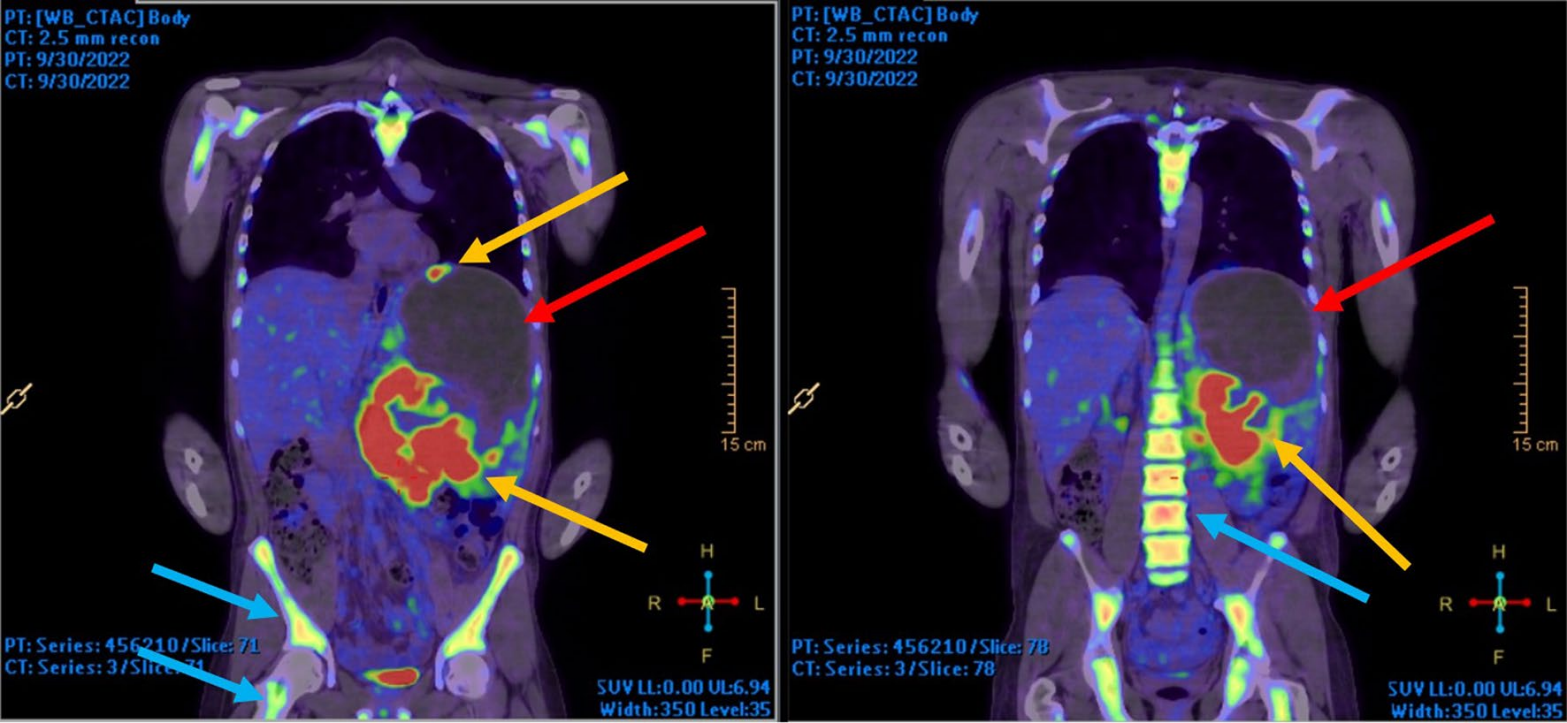
PET–CT images demonstrating tracer uptake in splenic periphery (orange arrows) and skeleton (blue arrows), as well as photopenic defects in the splenic core (red arrows)

A positron emission tomography (PET) scan was subsequently performed, demonstrated in Fig. 2. This showed a mixed density splenic mass with intense peripheral tracer uptake, particularly antero-superiorly and medially. In addition, it showed large photopenic defects in keeping with necrosis and diffusely increased bone marrow uptake throughout the axial and appendicular skeleton. The latter prompted further investigation with a trephine bone marrow core biopsy, which showed mild bone marrow hypercellularity with mild megakaryocytic atypia on histological analysis. This raised the possibility of an underlying myeloproliferative disease.

**Fig. 3:**
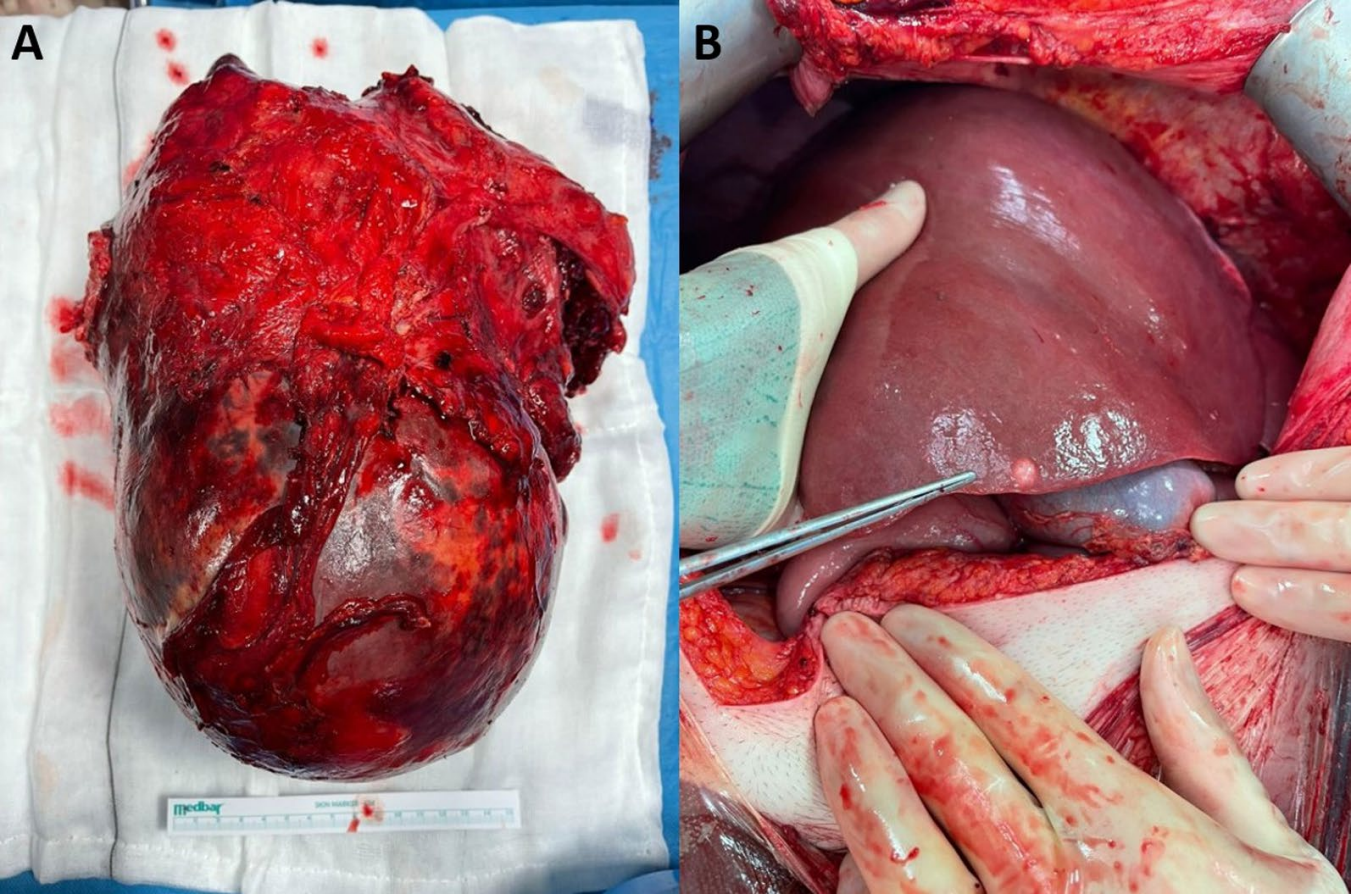
Intraoperative photographs showing resected splenic specimen (**A**) and one of the new liver lesions diagnosed intraoperatively (**B**)

Given that no definitive diagnosis was achieved at this stage, a diagnostic and therapeutic splenectomy was planned. Access to the abdominal cavity was achieved by an L-shaped laparotomy incision. A massive spleen adherent to the pancreatic tail at the hilum was identified and resected. In addition, three small liver lesions were detected in segment V, VII and VIII, respectively, as seen in Fig. 3 along with the specimen. Given the context, these were highly suspicious for metastatic deposits, but not present on magnetic resonance imaging performed 12 days prior. These small lesions along with the rest of the liver were assessed using intraoperative US. This deemed these three lesions as easily resectable and did not detect any further liver lesions. Therefore, an open splenectomy, distal pancreatectomy and liver metastasectomy were hence carried out.

Frozen section analysis on one of the metastatic liver deposits showed a well-circumscribed tumoural nodule comprising pleomorphic neoplastic cells with frequent bizarre nuclear forms. The findings were interpreted as a high-grade poorly differentiated malignancy, with lymphoma being the most likely diagnosis.

Gross pathological analysis showed a large splenic mass weighing 4 kg and measuring 28 cm, which was affixed to the pancreatic tail. On sectioning, the spleen was almost entirely replaced by a centrally cystically necrotic mass. The mass was largely non-viable however, towards the periphery, had a pale white, fleshy appearance. The tumour was centred on the spleen and did not appear to arise from the pancreas, with the resected pancreatic tail being unremarkable and free of malignancy. Analysis of the resected liver lesions demonstrated well-circumscribed fleshy white subcapsular tumour deposits, which were similar in morphology to the splenic mass.

**Fig. 4:**
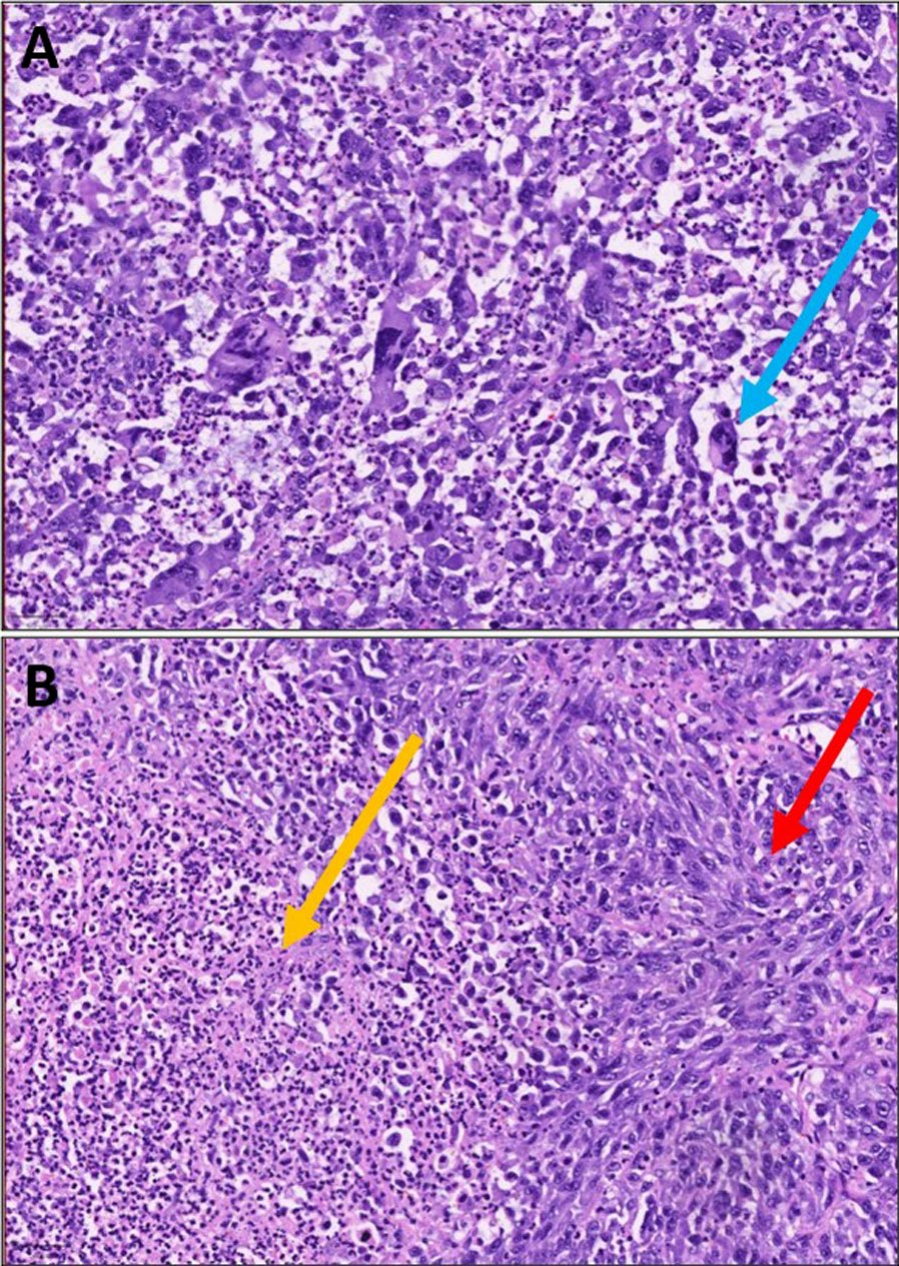
Histological slides showing **A** epithelioid component characterized by sheets of neoplastic cells with bizarre nuclei and atypical mitotic activity (blue arrow) and **B** spindled areas showing neoplastic cells organized in short fascicles (red arrow) and tumour necrosis (yellow arrow)—H&E × 200

Histological analysis of the splenic tumour showed diffuse effacement of the splenic parenchyma by a high-grade neoplasm. The tumour variably comprised neoplastic spindled and epithelioid cells. The spindle cell component was organized in short fascicles while the epithelioid component infiltrated the splenic parenchyma in cohesive sheets. The neoplastic cells were bizarre, pleomorphic and showed very brisk mitotic activity. Widespread areas of tumour necrosis were seen. The tumour invaded beyond the splenic capsule into the surrounding soft tissues. Slides demonstrating these findings are found in Fig. 4.

A broad immunohistochemical panel was applied. Cytokeratin stains (broad range cytokeratin, MNF116 and various other cytokeratins including cytokeratin 7, 20, 5/6 and 8/18) were negative, as were markers of melanocytic (S100, melan-A, SOX-10 and HMB-45), haemato-lymphoid/histiocytic (CD45, CD20, PAX5, CD2, CD3, CD5, CD56, CD68, CD138 and ALK1), dendritic cell (CD23, CD21 and CD123) and germ cell (PLAP, CD117, OCT3/4) differentiation. Immunohistochemical attempts at identifying a specific sarcomatoid lineage of differentiation were also negative, with vascular (CD31, ERG), neural (S100, SOX10) and adipocytic (S100, MDM2, CDK4) markers being negative. The tumour only showed non-specific expression of CD10, cyclin D1 and a high Ki67 proliferation fraction (around 80%).

The overall histological and immunohistochemical findings were thus supportive of a diagnosis of undifferentiated pleomorphic sarcoma (UPS) of the spleen, with metastatic deposits in the liver. The excision of both the splenic tumour and the hepatic metastatic deposits was deemed complete.

**Fig. 5:**
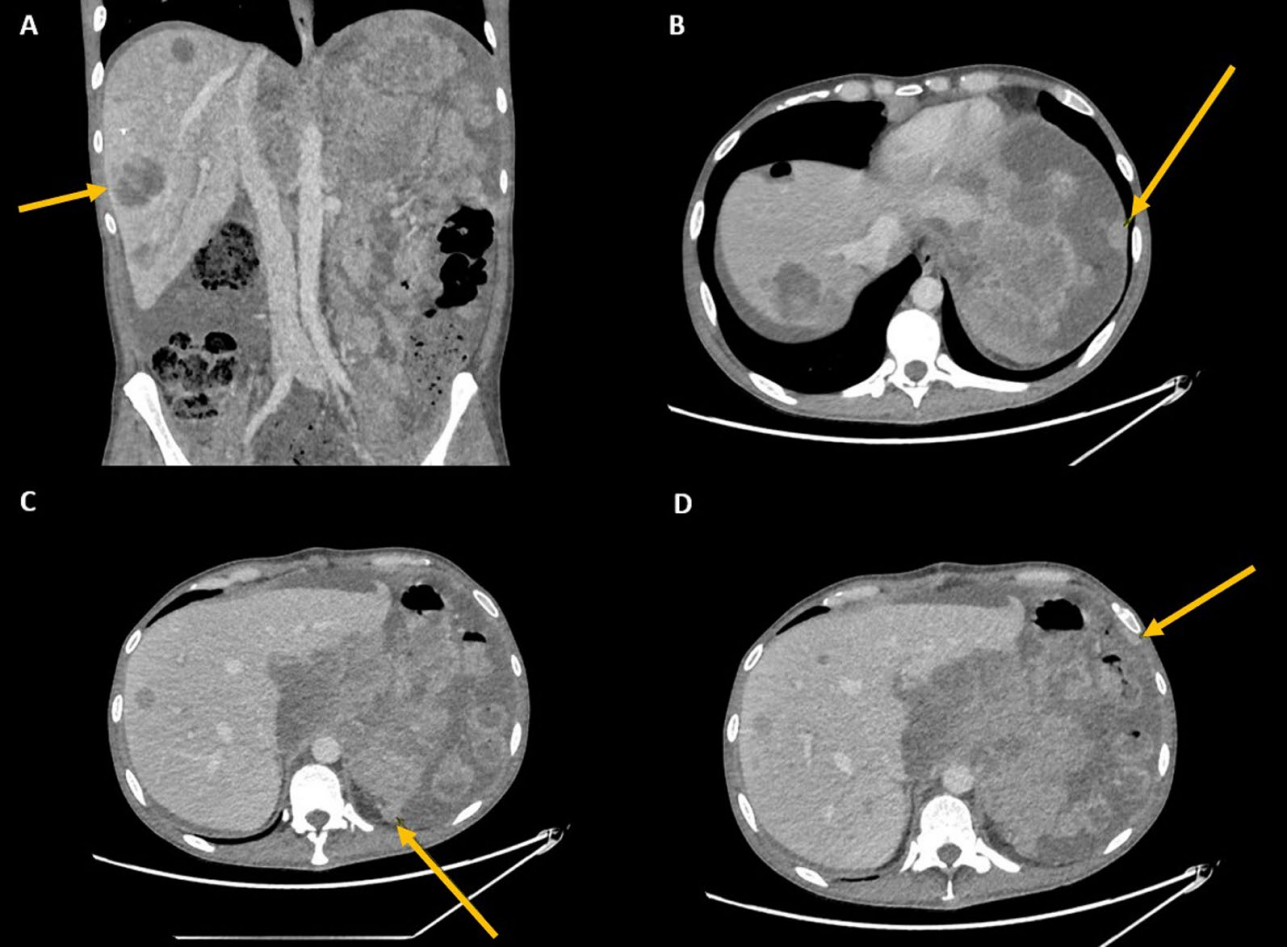
CT images demonstrating **A** extensive hepatic metastasis, **B** left subdiaphragmatic deposits, **C** splenic bed deposits and **D** parietal peritoneal deposits

The patient recovered well post operatively and was discharged home 11 days later, with a plan to start single-agent Doxorubicin at a starting dose of 30 mg/m^2^ weekly for 3 cycles when fully recovered from surgery. However, he presented to the Emergency Department yet again three weeks post operatively with persistent lower back and abdominal pain associated with fever. A CT scan of his abdomen was performed, showing extensive recurrent disease burden in the peritoneum and liver as seen in Fig. 5. He was hospitalized and given the first dose of doxorubicin the following day. The patient developed small bowel obstruction four days later, presumably secondary to tumour compression. Further chemotherapy was withheld in view of the patient’s poor condition and he eventually succumbed to his illness two and a half months after his initial presentation, and 34 days after splenectomy.

## Literature review and discussion

A literature search was performed using Medline, PubMed and Elsevier. 41 published cases were identified and are depicted in Table 1. Tumour or “cyst” size was not considered equivalent to spleen size so as to minimize bias. It was also used in preference to splenic weight due to the latter being underreported. The Response Evaluation Criteria in Solid Tumour (RECIST) criteria were used to grade the response to adjuvant chemoradiotherapy when this was part of patient management [1].

**Table 1:** Demographics, treatment modality, spleen size and initial adjuvant chemoradiotherapy response in all 41 cases identified within literature

First author	Year of publication	Age	Gender	Distant metastasis at diagnosis	Surgical management	Chemoradiotherapy	Spleen size	Initial response using RECIST evaluation
Govoni [10]	1982	51	F	No	Open splenectomy	n/a	21 × 25 × 10.5 cm	n/a
Wick [11] (Case No.1)	1982	48	M	Yes—liver	Open splenectomy	Unspecified adjuvant chemotherapy	N/S	n/s
Wick [11] (Case No.2)		51	F	No	Open splenectomy	n/a	N/S	n/a
Wick [11] (case No.3)		54	M	No	Open splenectomy	n/a	N/S	n/a
Jinno [12]	1987	53	M	Yes—liver, vertebrae, peritoneum	n/a	n/a	N/S	n/a
Bruneton [13]	1988	54	M	No	Open partial splenectomy	n/a	N/S	n/a
Sieber [14]	1990	41	M	Yes—omentum	Open splenectomy + omentectomy	Unspecified adjuvant chemoradiotherapy	28 × 17 × 12 cm	Progressive disease
Bonilla [15]	1994	42	F	No	Open splenectomy Surgical removal of lesions after recurrence	Adjuvant radiotherapy 5,300 cGy over surgical field + doxorubicin × 4 cycles	N/S	Progressive disease
Kimura [16] (Case No.1)	1998	29	M	No	Open splenectomy	Unspecified adjuvant chemoradiotherapy	17 × 13 × 8 cm	Progressive disease
Kimura [16] (Case No. 2)	1998	60	M	No	Open splenectomy	Adjuvant methylprednisolone Adjuvant cyclophosphamide, doxorubicin, vincristine, prednisolone (CHOP) × 2 cycles Adjuvant mitoxantrone, etoposide, carboplatin and prednisolone (MECP) × 1 cycle Adjuvant oral etoposide	8 × 8 × 8 cm	Progressive disease
Kimura [16] (Case No.3)	1998	66	F	No	Open splenectomy	Neoadjuvant prednisolone and gamma-globulins + spleen radiotherapy Adjuvant prednisolone + severe courses of different chemotherapy	N/S	Progressive disease
Mallipudi [17]	1998	73	F	No	Open splenectomy	n/a	15 × 10 × 8 cm	n/a
Gong [18]	1999	34	M	Yes—liver, omentum	Open splenectomy, distal pancreatectomy, retroperitoneal lymphadenectomy & liver biopsy	Adjuvant adriamycin × 3 days	20 × 16 × 10 cm	Progressive disease
Colovic [19]	2001	45	F	Yes—liver	Open splenectomy + liver biopsy	Adjuvant cyclophosphamide, doxorubicin, vincristine, prednisolone (CHOP) × 6 cycles	14 × 12 × 7 cm	Progressive disease^a^
Agha [20]	2002	57	F	No	Open splenectomy	n/a	10 × 10.7 × 9.5 cm	n/a
Audouin [21]	2003	71	F	No	Open splenectomy	Unspecified adjuvant chemotherapy	14 × 12 × 10 cm	Progressive disease^b^
Ozaras [22]	2003	51	F	No	Open splenectomy	n/a	N/S	n/a
Moloney [23]	2004	19	F	No	Emergency open splenectomy	Adjuvant doxorubicin and ifosfamide × 2 cycles	22 × 17 × 13 cm	Progressive disease
Katsuura [24]	2006	82	M	No	Open splenectomy	n/a	13 cm maximal diameter	n/a
Low [25]	2006	63	F	Yes—liver, lung, myocardial	n/a	n/a	N/S	n/a
Koboyashi [26]	2008	82	F	Yes—liver	n/a	Palliative radiotherapy—10 Gy in 10 fractions to the spleen over 2 weeks	19 × 13 × 9 cm	Progressive disease
Oka [27]	2008	58	F	No	Open splenectomy	n/a	14.5 × 8.5 × 9.5 cm	Progressive disease
Hashmi [7]	2010	76	M	No	Laparoscopic splenectomy	n/a—patient refused adjuvant treatment	N/S	n/a
Amatya [28]	2011	77	M	Yes—renal, adrenal, femur	n/a	n/a	N/S	n/a
Ji-Feng [29]	2011	48	F	No	Laparoscopic splenectomy + fenestration of hepatic cysts	n/a	11 × 10 × 8 cm	n/a
Liang He [30]	2011	35	M	No	Emergency open splenectomy	n/a	13 × 11 × 8 cm	n/a
Gupta [31]	2012	30	M	No	Open splenectomy	n/a—patient failed to turn up after hospital discharge	15 × 12 × 6 cm	n/a
Yamada [32]	2012	67	F	No	Open splenectomy	n/a	14.5 × 12 × 8 cm	n/a
Yamamoto [33]	2012	69	M	Yes—liver	Open splenectomy	Neoadjuvant paclitaxel, carboplatin and etoposide × 2 cycles Adjuvant mitomycin C and etoposide via hepatic arterial infusion × 6 cycles	N/S	Complete response
Das [34]	2013	30	M	Yes—liver	Open splenectomy + deroofing of hepatic cysts + liver biopsy	n/a	N/S	n/a
Rakic [35]	2013	57	M	No	Laparotomy—removal of large solitary mass in left upper abdomen, partial gastrectomy, partial resection of left crus of diaphragm, distal pancreatectomy, left hemicolectomy	n/a	N/S	n/a
Yamamoto [33]	2013	81	F	No	Laparoscopic splenectomy	n/a	21 × 12 × 5 cm	n/a
Batra [36]	2016	17	M	No	Open splenectomy	Adjuvant cyclophosphamide, doxorubicin, vincristine, prednisolone (CHOP) × 3 cycles Adjuvant ifosfamide, carboplatin and etoposide (ICE) × 3 cycles Autologous stem cell transplant using carmustine, etoposide, cytarabine and melphalan (BEAM)	24 × 14 × 11 cm	Complete response
Makis [37]	2017	63	M	Yes—liver	n/a	n/a	N/S	n/a
Mantas [9]	2016	66	F	No	Open splenectomy	n/a	18 × 13 × 10 cm	n/a
Ashmoore [38]	2020	56	F	No	Open splenectomy	n/a	15 cm maximal diameter	n/a
Huang [39]	2020	40	F	No	Laparoscopic splenectomy	Neoadjuvant prednisolone	12 × 8 × 3.6 cm	n/a
Darwish [40]	2022	49	n/s	Yes—lung	Delayed elective open splenectomy	Adjuvant doxorubicin and ifosfamide × 3 cycles	16 cm (craniocaudal)	Progressive disease
Koboyashi [41]	2022	61	F	No	Open splenectomy	n/a	19 × 13 × 9 cm	n/a
Luo [44]	2022	60	F	No	Laparoscopic splenectomy	Adjuvant oral imatinib	N/S	Complete response
Wijebandara [42]	2022	52	F	No	Open splenectomy	n/a	N/S	n/a

^a^Initial good response not considered as it was based on ultrasound findings

^b^“Good initial clinical results” not considered as it was not based on any imaging or tumour markers

Splenic UPS in an extremely rare entity with only 41 other cases reported within English literature at the time of writing this report. UPS pathologically defines a sarcoma subtype lacking any specific lineage differentiation and remains a diagnosis of exclusion after other pleomorphic sarcoma subtypes have been excluded. Whilst still in use, the term ‘malignant fibrous histiocytoma’ has fallen out of favour in recent WHO classifications as the tumour does not show clear evidence of fibrohistiocytic differentiation. UPS may be divided into five histological subtypes which are pleomorphic, inflammatory, myxoid, giant cell and angiomatoid [8, 29]. However, despite these subtypes being present, there appears to be no difference in prognosis between them [19].

This first case of splenic UPS was described by Govoni et al. [10, 29]. They commonly present with left upper quadrant pain, weight loss and concurrent anaemia. In the majority of cases, a short disease history is observed, with only a few having long standing manifestations [22, 40]. In three cases, the patients presented with splenic rupture associated with haemodynamic compromise [28, 30, 40]. The subject case also presented with a non-specific one-week history of abdominal pain without associated symptomatology or biochemical manifestations [7, 10, 14, 17, 19, 22, 25–27, 30, 33, 35, 37, 38, 41, 42].

The age at presentation ranges from 17 to 82 [36, 41]. There appears to be no particular gender predilection. In most cases, including the subject case, the patients were previously healthy with no specific comorbidities. The case by Agha et al. presented on a background of systemic amyloidosis with the patient developing nephrotic syndrome after tumour removal [20]. Given the rarity of the diagnosis, the disease is rarely suspected on radiological or clinical grounds, with the most cases suspecting lymphoma at the first instance. As presented here, most cases only reached the final diagnosis after thorough histopathological work up.

The histopathological features are similar to conventional UPS in soft tissues. The histological appearance is by no means distinctive but is often a combination of spindled, epithelioid and bizarre pleomorphic multinucleated cells exhibiting brisk mitotic activity, with or without tumour necrosis. Osteoclast-like giant cells are typically noted in the ‘giant cell’ variant, while a brisk inflammatory infiltrate is typically noted in the ‘inflammatory’ variant. A myxoid stroma background may be seen and is typically extensive in the ‘myxoid’ variant, making differentiation with high-grade myxofibrosarcoma challenging. A pseudovascular configuration may be noted in the 'angiomatoid’ variant, with differentiation from angiosarcoma being established through the absence of vascular marker expression on immunohistochemical grounds. The latter serves to distinguish UPS from somewhat commoner splenic sarcomas including splenic angiosarcoma, Kaposi sarcoma and littoral cell angiosarcoma.

The immunohistochemical profile of UPS is non-specific and is principally geared at excluding other diagnoses. The tumour is persistently negative for cytokeratins and markers of melanocytic differentiation. Non-specific expression of lysozyme, CD68, fascin, CD10 and cyclin D1 may be observed. However, expression of histiocytic markers is only focal which helps to differentiate this tumour from histiocytic sarcoma.

Due to the paucity of data and rare nature of the disease, there are no established staging criteria for splenic UPS. This has implications on stratifying patients and guiding management [8, 9]. The most commonly used imaging modalities were computed tomography or ultrasonography. The latter was frequently used for guiding biopsies. Computed tomography often demonstrated a heterogeneous splenic cyst with peripheral enhancement on computed tomography [7, 31, 40].

Response to adjuvant treatment was generally poor, with only three papers reporting a positive response. The initial complete response quoted in Colovic et al. was based on the complete disappearance of all liver lesions on ultrasound. This form of imaging is not recommended by the latest RECIST criteria which recommend cross-sectional imaging for assessing treatment response. In addition, the good initial response quoted by Audouin et al. was not supported by any objective measures such as imaging or tumour markers. Hence, the adjuvant treatment in both of these papers was not deemed to have any positive response using the RECIST criteria [43], prior to the eventual disease progression a few months later. None of the chemoradiotherapy regimens stood out as superior when factoring response.

Table 2 classifies the included case reports by patient survival. Ashmoore et al., Ozaras et al. and Hashmi et al. were excluded from this survival analysis as patient survival was not specified within the report. Gupta et al. was also similarly excluded as the patient was lost to follow-up, whilst Govoni et al. was excluded as the patient was not followed up after 7 months.

**Table 2:** Case reports by reported survival

Reported survival from presentation	< 1 year	1–2 years	> 2 years
Papers	Wick et al. [11]—Case 1 Jinno et al. [12] Bruneton et al. [13] Sieber et al. [14] Bonilla et al. [15] Gong et al. [18] Audouin et al. [21] Moloney et al. [23] Low et al. [25] Koboyashi et al. [26] Oka et al. [27] Amatya et al. [28] Liang He et al. [30] Yamada et al. [32] Das et al. [34] Rakic et al. [35] Yamamoto et al. [33] Batra et al. [36] Makis et al. [37] Huang et al. [39] Wijebandara et al. [42]	Wick et al. [11]—Case 2 and 3 Kimura et al. [16]—Case 2 and 3 Mallipudi et al. [17] Colovic et al. [19] Agha et al. [20] Katsuura et al. [24] Ji-Feng et al. [29] Luo et al. [44] Kobayashi et al. [41]	Kimura et al. [16]—Case 1 Yamamoto et al. [33] Mantas et al. [9] Darwish et al. [40]

Table 3 demonstrates the average spleen maximal diameter classified by these survival groups. Papers that failed to report this were excluded from this analysis. Survivors under one year had larger spleens compared to survivors over one year. This suggests that a larger spleen size is a risk factor for poorer prognosis.

**Table 3:** Average spleen maximal diameter by patient survival group

Patient survival	Average spleen maximal diameter (cm)
< 1 year	17.41
1–2 years	12.95
> 2 years	12.85

Table 4 demonstrates patient survival by metastasis and splenectomy status. 12 of 41 cases (29.3%) were found to have distant metastasis on initial diagnosis, with splenectomy being performed in 7 of these cases. The presence of metastasis on diagnosis impacted survival, with 75% of patients with metastasis surviving less than one year compared with 50% of patients without metastasis. Only 3 patients (25%) with metastatic disease on diagnosis survived longer than one year, all of which underwent splenectomy and adjuvant chemotherapy.

**Table 4:** Patient survival by metastasis and splenectomy status

Patient group		Patients with recorded survival	Survival, *n* (%)		
			< 1 year	1–2 years	> 2 years
All patients	Total	36	21 (58.3)	11 (30.6)	4 (11.1)
	Splenectomy	31	16 (51.6)	11 (35.5)	4 (12.9)
	No splenectomy	5	5 (100)	0	0
No metastasis present	Total	24	12 (50)	10 (41.7)	2 (8.3)
	Splenectomy	24	12 (50)	10 (41.7)	2 (8.3)
	No splenectomy	0	0	0	0
Metastasis present	Total	12	9 (75)	1 (8.3)	2 (16.7)
	Splenectomy	7	4 (57.1)	1 (14.3)	2 (28.6)
	No splenectomy	5	5 (100)	0	0

As the preoperative imaging in our case did not demonstrate evidence of metastatic disease, surgery was performed with curative intent. As previously described, these three sole liver lesions were easily resectable and so the decision was made to proceed with surgery. In addition, the removal of such a large 4 kg tumour relieved its burden, including discomfort and early satiety. Other than a few select cases from the literature review, surgical resection in established metastatic disease did not appear to offer much survival benefit, including in the presented case. Hence, the presence of metastasis should generally be considered as a contra-indication to surgical resection.

The four cases with a reported survival of more than two years differed greatly in patient characteristics and management. Kimura et al. report a case of a 29-year-old male who underwent elective splenectomy four months after diagnosis. The spleen size was 17 cm × 13 cm × 8 cm and weighed 735 g. No metastases were detected before or during surgery and he did not receive any chemotherapy or radiotherapy. The patient subsequently developed liver metastases 3 years after surgery and a liver metastasectomy was performed, followed by an unspecified regime of adjuvant chemoradiotherapy. Out of all the included cases, this was the longest documented survivor at 5 years from presentation.

Mantas et al. report a 66-year-old lady who underwent immediate splenectomy following diagnosis. The splenic size was similar to the prior case at 18 cm × 13 cm × 10 cm. However, the splenic weight was almost double at 1300 g. Similarly, no metastases were detected before or during surgery and she did not receive any chemotherapy or radiotherapy. She was reported as alive, asymptomatic and free of local recurrence and metastatic disease three and a half years after her operation.

Darwish et al. report a 49-year-old gentleman who had initially refused treatment. Imaging had shown multiple splenic lesions with the largest being 11 cm × 9 cm × 10 cm. However, the patient presented eight months later with splenic rupture which was initially treated conservatively. The patient then underwent elective splenectomy 11 months after initial presentation, after which he received three cycles of doxorubicin and ifosfamide. New liver lesions were later detected on follow-up imaging for which the patient underwent microwave ablation (MWA) and a left hepatectomy. Despite post-operative recurrence, the authors report that the patient was still alive 2 years following presentation.

Lastly, Yamamoto et al. report a 69-year-old male who presented with a splenic mass measuring 4.5 cm × 5.4 cm × 5 cm with liver lesions. The patient had a good response to two cycles of neoadjuvant carboplatin, etoposide and paclitaxel, after which he underwent elective splenectomy. Following recovery, he received six cycles of mitomycin and etoposide via hepatic arterial infusion (HAI). He was reported alive three and a half years following presentation.

Therefore, survival was not found to be associated with any specific patient characteristic or management strategy.

## Conclusion

UPS of the spleen is a malignancy characterized by pleomorphic spindle cells with bizarre looking nuclei, arranged in a storiform pattern. It is generally treated by splenectomy and in some instances chemotherapy. The disease carries an overall dismal prognosis, particularly when combined with distant metastasis. Currently available literature fails to delineate a particularly advantageous or superior management strategy which increases survival.

## Data Availability

All data in this report were obtained from our institution’s internal patient investigation and imaging software (iSOFT and PACS, respectively). All data generated or analysed during this study are included in this published article.

## Abbreviations

STS: Soft tissue sarcoma
UPS: Undifferentiated pleomorphic sarcoma
MFH: Malignant fibrous histiocytoma
WCC: White cell count
CRP: C-reactive protein
US: Ultrasound
CT: Computed tomography
MR: Magnetic resonance
PET: Positron emission tomography
MWA: Microwave ablation
HAI: Hepatic arterial infusion
